# Indole Glycosides from Aqueous Fraction of *Strychnos nitida*

**DOI:** 10.1007/s13659-016-0112-8

**Published:** 2016-11-09

**Authors:** Bei Wang, Zhi Dai, Lu Liu, Xin Wei, Pei-Feng Zhu, Hao-Fei Yu, Ya-Ping Liu, Xiao-Dong Luo

**Affiliations:** 1 State Key Laboratory of Phytochemistry and Plant Resources in West China, Chinese Academy of Sciences, Kunming, 650201 China; 2 Key Laboratory of Animal Models and Human Disease Mechanisms, Kunming Institute of Zoology, Chinese Academy of sciences, Kunming, 650223 Yunnan China; 3 University of Chinese Academy of Sciences, Beijing, 100049 China; 4 Yunnan Key Laboratory of Natural Medicinal Chemistry, Chinese Academy of Sciences, Kunming, 650201 China

**Keywords:** *Strychnos nitida*, Indole glycosides, Alkaloids

## Abstract

Three new indole glycosides 22-deoxystrictosamide (**1**), 22-deoxystrictosamide *N* ^b^-oxide (**2**) and vincosamide 2′-*O*-*β*-D-xylopyranoside-11-*O*-*β*-D-glucopyranoside (**3**), together with four known analogues were isolated from aqueous fraction of *Strychnos nitida*. Their structures were elucidated on the basis of extensive analysis of spectroscopic data. All the alkaloids were tested for their cytotoxic activity, but they did not show any exciting result.

**Electronic supplementary material:**

The online version of this article (doi:10.1007/s13659-016-0112-8) contains supplementary material, which is available to authorized users.

## Introduction

Monoterpenoid indole alkaloids comprising of over 3000 natural alkaloids derived from condensation of tryptamine and secologanin [1]. Many of them, such as yohimbine [2], reserpine [3], and camptothecin [4] are well known for their pharmacological significance. In our continual searching for antitumor natural products, many cytotoxic indoles and bisindoles with novel structures were isolated [5–16]. *Strychnos* *nitida* G. Don (Loganiaceae) is a medicinal plant indigenous to Yunnan province, China. Previous investigations focused on the non-polar indoles with different skeletons, which were responsible for their medicinal properties, especially the remarkable strychnine and brucine [17, 18]. The polar indole alkaloids in aqueous fraction of medicinal plants were always neglected, which inspired us to carry out phytochemical investigation on aqueous fraction of *S.* *nitida.* As a result, three new indole glycosides, 22-deoxystrictosamide (**1**), 22-deoxystrictosamide *N* ^b^-oxide (**2**) and vincosamide 2′-*O*-*β*-D-xylopyranoside-11-*O*-*β*-D-glucopyranoside (**3**), together with four known analogues vincosamide (**4**) [19], antirhine *β*-methochloride (**5**) [20], 3-*epi*-strictosidinic acid (**6**) [21], vincoside (**7**) [22] were isolated. All alkaloids (**1**–**7**) were evaluated for their cytotoxic activity, but none of them showed exciting result against five human cancer cell lines (T98G, U87, A549, GITC-3#, and GITC-18#), though the various bioactivities of the chemical constituents from S*trychnos* were reported previously [23–27].

## Results and discussion

**Fig. 1:**
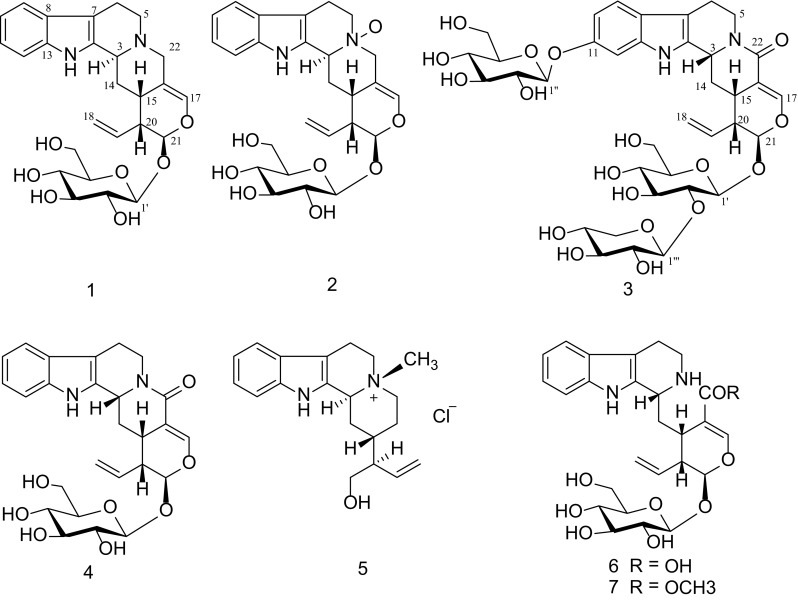
Structures of alkaloids **1–7**

**Fig. 2:**
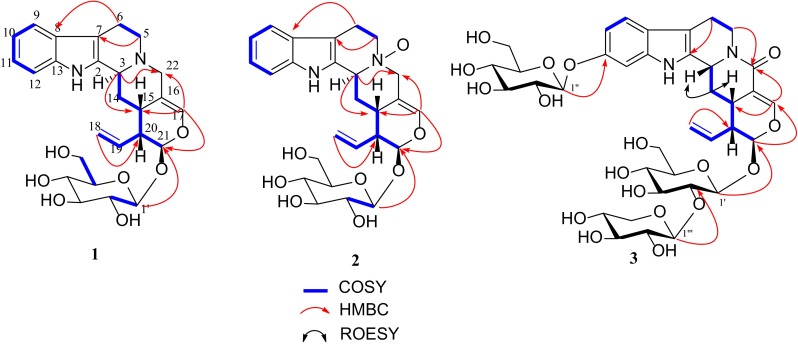
^1^H-^1^H COSY, ROESY and HMBC key correlations of alkaloids **1–3**

Compound **1** was deduced to have a molecular formula of C_26_H_32_N_2_O_7_, as indicated by the observed ion peak at m/z 485.2283 [M + H] ^+^ (calcd. for 485.2282) in its HRESIMS data, indicating 12 indices of hydrogen deficiency. The ^1^H NMR spectrum exhibited four aromatic proton signals assignable to an ortho-substituted benzene moiety [*δ*_H_ 7.40 (1H, d, *J* = 8.0 Hz, H-9), 7.00 (1H, ddd, *J* = 8.0,7.1,1.0 Hz, H-10), 7.08 (1H, ddd, *J* = 8.0,7.1,1.0 Hz, H-11), and 7.34 (1H, d, *J* = 8.0, H-12)], a typical indole aromatic moiety (Table 1), which were in agreement with the carbon signals at *δ*_C_ 118.6 (d, C-9), 119.9 (d, C-10), 122.1 (d, C-11), and 112.1 (d, C-12), supported by the HSQC experiment. The ^1^H and ^13^C NMR data (Tables 1 and 2) of **1** exhibited high similarities with those of strictosamide [19], except for a carbonyl group (*δ*_C_ 166.0) replaced by a methylene group (*δ*_C_ 46.6; *δ*_H_ 3.45, 3.00) in **1**. Comparing the ^1^H and ^13^C NMR spectral data of two compounds, assumed the reduction of the carbonyl at C-22 in compound **1** by deshielded signal for C-5. This assumption was further supported by the correlation of *δ*_H_ 4.47 (H-3) and 6.22 (H-17) with *δ*_C_ 46.6 (C-22) in the HMBC spectrum. H-15 and H-20 of *β*-orientation and H-21 of *β*-orientation were derived from the iridiod secologanin. In addition, The ROESY correlations of H-3 (*δ*_H_ 4.47, br s) with one of H-14 (*δ*_H_ 1.91, td, *J* = 14.0, 5.1 Hz), H-15 (*δ*_H_ 2.47, m) with another H-14 (*δ*_H_ 2.32, ddd, *J* = 14.0, 5.1, 2.4 Hz) showed that H-3 and H-15 were located on the opposite side. Detailed analysis of 2D NMR spectroscopic data of **1** (Fig. 2) suggested that its other parts were the same to those of strictosamide. Hence, the structure of **1** was elucidated to be 22-deoxystrictosamide (Fig. 1).

**Table 1:** The ^1^H NMR and ^13^C NMR data assignments for the aglycones of **1**–**3 (**methanol-*d*_4_ *δ* in ppm**)**

Position	**1**		**2**		**3**	
	*δ*_H_ (*J* in Hz) (400 M)	*δ*_C_ (125 M)	*δ*_H_ (*J* in Hz) (500 M)	*δ*_C_ (125 M)	*δ*_H_ (*J* in Hz) (400 M)	*δ*_C_ (150 M)
**2**		132.9		130.4		133.3
**3**	4.47, br. s	55.1	4.68, br. s	71.3	4.94, d (11.2)	55.1
**5a**	3.27, overlap	51.7	3.77, m	68.9	5.06, dd (12.4, 4.0)	41.5
**5b**					2.99, td (12.4, 3.0)	
**6a**	2.60, m	17.6	3.14-3.05, m	21.0	2.91, m	24.0
**6b**	3.08, m				3.32, overlap	
**7**		107.4		106.6		109.0
**8**		128.8		127.8		118.7
**9**	7.40, d (8.0)	118.6	7.45, d (8.0)	119.1	7.00, d (8.6)	123.2
**10**	7.00, ddd (8.0, 7.1, 1.0)	119.9	7.05, ddd (8.0, 7.1, 1.0)	120.7	6.74, dd (8.6, 4.1)	104.4
**11**	7.08, ddd (8.0, 7.1, 1.0)	122.1	7.14, ddd (8.0, 7.1, 1.0)	123.3		153.3
**12**	7.34, d (8.0)	112.1	7.37, d (8.0)	112.6	7.00, d (4.1)	106.8
**13**		137.8		138.9		139.9
**14a**	1.91, td (14.0, 5.1)	29.9	2.23, ddd (14.1, 4.8, 1.6)	25.5	1.47, q (13.0)	32.5
**14b**	2.32, ddd (14.0, 5.1, 2.4)		2.69, overlap		2.48, dt (13.0, 3.6)	
**15**	2.47, m	25.5	2.50, m	24.5	3.23, overlap	27.6
**16**		112.9		107.9		109.6
**17**	6.22, s	134.7	6.24, s	138.7	7.43, d (2.0)	149.0
**18a**	5.27, overlap	119.1	5.28, overlap	119.5	5.23, d (10.3)	120.5
**18b**	5.32, d (1.8)		5.31, d (1.8)		5.33, d (17.0)	
**19**	5.82, dt (17.2, 10.2)	136.8	5.94, dt (17.2,10.2)	136.3	5.57, dt (17.2, 10.1)	133.9
**20**	2.69, m	46.6	2.68, overlap	46.4	2.73, dd (6.1, 2.2)	44.5
**21**	5.27, d (2.3)	97.2	5.28, d (1.8)	97.4	5.49, d (2.0)	97.2
**22a**	3.45, d (12.9)	46.6	4.18, d (13.2)	60.1		166.0
**22b**	3.01, overlap		3.34, s

Compound **2** exhibited a molecular ion peak at m/z 500.2150 (calcd. for 500.2159) in its HREIMS spectrum, indicating the molecular formula of C_26_H_32_N_2_O_8_, sixteen mass units higher than that of **1**. Its ^1^H NMR spectrum revealed four downfield shifts signal at *δ*_H_ 4.68 (H-3), *δ*_H_ 3.77 (H-5) and *δ*_H_ 4.18 and 3.34 (H-22), while the ^13^C NMR data exhibited noticeable downfield shifts involving *δ*_C_ 71.3 (C-3), *δ*_C_ 68.9 (C-5) and *δ*_C_ 60.1 (C-22) in **2** comparison to those of **1**. These features are characteristic of *N* (4)-oxides [28, 29], and which was further supported by the HMBC correlations of *δ*_H_ 3.77 (H-5), 2.50 (H-15), and 4.18 (H-22) with *δ*_C_ 71.3 (d, C-3). The ROESY correlations indicated that the relative configuration of **2** was the same as that of **1**. Besides, other parts of **2** were identical to those of **1** as supported by detailed analysis of extensive 2D NMR spectral data of **2** (Fig. 2). Thus, the structure of **2** was elucidated as 22-deoxystrictosamide *N* ^b^-oxide (Fig. 1).

Compound **3**, Its molecular formula was deduced as C_37_H_48_N_2_O_18_ based on ^13^C NMR and HRESIMS data (m/z 831.2791 [M + Na]^+,^ calcd. for 831.2794). The ^1^H and ^13^C NMR spectra of **3** resembled those of vincosamide 11-*O*-*β*-D-glucopyranoside [22], but exhibited for more one pentosyl moiety. Acid hydrolysis of **3** produced D-xylose and D-glucose as sugar residues, which were determined by GC analysis of their corresponding trimethylsilylated L-cysteine adducts. The coupling constants of the anomeric protons [*δ*_H_ 4.84 (d, *J* = 8.0 Hz, H-1′), 5.11 (d, *J* = 7.8 Hz, H-1″) and 4.48 (d, *J* = 7.4 Hz, H-1‴)] suggested *β*-pyranosyl configuration for both D-xylose and D-glucose moieties. Since NMR signals of three monosaccharides overlapped undesirablely, The HSQC–TOCSY allowed all of the carbons belonging to each sugar moiety. In particular, a first spin system constituted by protons linked to six carbons at *δ*_C_ 97.7, 82.3, 77.9, 71.4, 78.2, and 62.6, the second represented by *δ*_C_ 102.0, 75.2, 78.1, 71.4, 78.6, and 62.6 and finally the third originated by *δ*_C_ 106.2, 75.9, 77.6, 71.2, 67.3 (in good accordance with the presence of xylose moiety) were evident (Table 2) [30]. The additional xylosyl moiety was positioned at C-2′ by the HMBC correlation between *δ*_H_ 4.48 (H-1‴) and *δ*_C_ 82.3 (C-2′). Besides one more xylosyl, the ROESY correlations were differ from **1** and **2**, which showed H-3 (*δ*_H_ 4.94, d, *J* = 11.2 Hz) with H-15 (3.22, m) were cofacial, so H-3 was *β*-orientation. The planner structure was identical to those of vincosamide 11-*O*-*β*-D-glucopyranoside [22] as supported by intensive analysis of its 2D NMR spectral data (Fig. 2). Then, the structure of **3** was elucidated to vincosamide 2′-*O*-*β*-D-xylopyranoside-11-*O*-*β*-D-glucopyranoside (Fig. 1).

**Table 2:** The NMR data assignments for the sugar moieties of **1–3 (**methanol-*d*_4_ *δ* in ppm**)**

Position	**1**		**2**		**3**	
	*δ*_H_ (*J* in Hz)	*δ*_C_	*δ*_H_ (*J* in Hz)	*δ*_C_	*δ*_H_ (*J* in Hz)	*δ*_C_
**1′**	4.55, d (8.0)	99.4	4.51, d (8.0)	99.6	4.84, d (8.0)	97.7
**2′**	3.01, overlap	74.4	2.97, dd (8.9, 8.0)	74.5	3.45, t (8.0)	82.3
**3′**	3.28, overlap	78.0	3.22, overlap	78.0	3.61, t (8.7)	77.9
**4′**	3.24, overlap	71.5	3.19, overlap	71.4	3.37, overlap	71.4
**5′**	3.25, overlap	78.1	3.21, overlap	78.2	3.49, overlap	78.2
**6′a**	3.65, dd (11.9, 5.5)	62.6	3.62, dd (11.9, 5.0)	62.6	3.70, m	62.6
**6′b**	3.87, dd (11.9, 1.9)		3.84, dd (11.9, 1.9)		3.93, br. d (12.3)	
**1″**					5.11, d (7.8)	102.0
**2″**					3.56, t (7.8)	75.2
**3″**					3.34, overlap	78.1
**4″**					3.43, overlap	71.4
**5″**					3.50, overlap	78.6
**6″a**					3.72, m	62.6
**6″b**					3.93, br. d (12.3)	
**1′′′**					4.48, d (7.4)	106.2
**2′′′**					3.22, overlap	75.9
**3′′′**					3.30, t (8.9)	77.6
**4′′′**					3.42, overlap	71.2
**5′′′a**					3.79, dd (11.4, 5.3)	67.3
**5′′′b**					3.14, t (11.2)	

## Experimental Section

### General Experimental Procedures

Optical rotations were obtained with a Jasco P-1020 Automatic Digital Polariscope. UV spectra were measured with a Shi madzu UV2401PC spectrometer. IR spectra were obtained on a Bruker FT-IR Tensor-27 infrared spectrophotometer with KBr pellets. ^1^H, ^13^C, and 2D NMR spectra were recorded on a Bruker DRX-400 NMR, Bruker DRX-500 NMR and Bruker DRX-600 spectrometer with TMS as internal standard. ESI-MS and HR-EI-MS analysis were carried out on Waters Xevo TQS and Waters AutoSpec Premier P776 mass spectrometers, respectively. Semi-preparative HPLC was performed on a Waters 600 HPLC with a COSMOSIL 5C_18_ MS-II (10ID × 250 mm) column. Silica gel (100–200 and 200–300 mesh, Qingdao Marine Chemical Co. Ltd., P.R. China), Sephadex LH-20 (GE Healthcare Bio-Xciences AB), RP-18 gel (20–45 μm, Fuji Silysia Chemical Ltd., Japan), and MCI gel (75–150 μm, Mitsubishi Chemical Corporation, Tokyo, Japan) were used for column chromatography. Fractions were monitored by TLC (GF 254, Qingdao Marine Chemical Co., Ltd., Qingdao), and spots were visualized by Dragendorff’s reagent.

### Plant Material

Air-dried twigs of *S. nitida* were collected in November 2006 from Xishuangbanna, Yunnan province, P. R. China. The plant was identified by Mr. Jing-Yun Cui, Xishuangbanna Tropical Botanical Garden. Chinese Academy of Sciences. A voucher specimen (No. Luo20060412) has been deposited at the State Key Laboratory of Phytochemistry and Plant Resources in West China, Kunming Institute of Botany, Chinese Academy of Sciences.

### Extraction and Isolation

The air-dried and powdered twigs of *S. nitida* (7.0 kg) were extracted with MeOH under reflux conditions, and the solvent was evaporated in vacuo. The residue was dissolved in 0.37% HCl (pH 2–3) and the solution was subsequently basified using 10% ammonia to pH 9–10. The basic solution was partitioned with EtOAc, affording a two-phase mixture. The EtOAc fraction (40 g) and H_2_O fraction (100 g). Then H_2_O fraction (100 g) was subjected to a macroporous resin D101 and eluted with MeOH/H_2_O system to give MeOH fraction (28 g). The MeOH fraction (28 g) was separated by silica gel column chromatography (CC), eluted with CHCl_3_/MeOH/H_2_O (10:1:0.1, 8:2:0.2, 7:3:0.5, 6:4:1, v/v/v) to give four subfractions (Fr. A-D) and **7** (2.733 g). Fr. C (5.2 g) was separated on a Sephadex LH-20 column eluting with MeOH, to obtain subfraction C1 and C2. Subfraction C1 was then separated by RP-18 MPLC (MeOH/H_2_O, 8:92 to 60:40) and semipreparative HPLC (MeCN/H_2_O, 20:80) to yield **1** (30.0 mg) and **4** (5.6 mg), Subfraction C2 was subjected to RP-18 CC (MeOH/H_2_O, 10:90 to 50:50) and then purified by semipreparative HPLC (MeCN/H_2_O, 25:75) to afford alkaloids **5** (10.6 mg) and **6** (5.1 mg). Fr. D (7.2 g) was chromatographed on macroporous resin MCI, Sephadex LH-20 and Semi-preparative HPLC successively to afford **2** (36.1 mg) and **3** (2.3 mg).

#### *22*-*Deoxystrictosamide* (**1**)

Yellowish amorphous powder, [*α*]_*D*_^25^−79.2 (*c* 0.15, MeOH); UV (MeOH) λ_max_ (log *ε*) nm 225 (4.56), 209 (4.43), 281 (3.91); IR (KBr) *ν*_max_ 3423, 2923, 1450, 1317, 1238, 1138, 1075, 1013, 928, 743 cm^−1^; ^1^H, ^13^C-NMR spectroscopic data see Table 1; ESIMS m/z 485 [M + H]^+^; HRESIMS m/z 485.2283 [M + H]^+^ (calcd for C_26_H_32_N_2_O_7_, 485.2282).

#### *22*-*Deoxystrictosamide N*^*b*^-*oxide* (**2**)

Yellowish amorphous powder, [*α*]_*D*_^25^−99.5 (*c* 0.10, MeOH); UV (MeOH) λ_max_ (log *ε*) nm 221 (4.62), 274 (3.88); IR (KBr) *ν*_max_ 3425, 2923, 1454, 1384, 1239, 1143, 1073, 1012, 935, 744 cm^−1^; ^1^H, ^13^C-NMR spectroscopic data see Table 1; ESIMS m/z 501 [M + H]^+^; HREIMS m/z 500.2150 [M]^+^ (calcd for C_26_H_32_N_2_O_18_, 500.2159).

#### *Vincosamide 2′*-*O*-*β* -*D*-*xylopyranoside*-*11*-*O*-*β* -*D*-*glucopyranoside* (**3**)

Pale-yellow amorphous powder, [*α*]_*D*_^25^−117.7 (*c* 0.10, MeOH); UV (MeOH) λ_max_ (log *ε*) nm 226 (4.55), 199 (4.31); IR (KBr) *ν*_max_ 3442, 2924, 1633, 1432, 1383, 1248, 1169, 1073, 876, 598 cm^−1^; ^1^H, ^13^C-NMR spectroscopic data see Table 1; ESIMS m/z 809 [M + H]^+^; HRESIMS m/z 831.2791 [M + Na]^+^ (calcd for C_37_H_48_N_2_O_18_, 831.2794).

### Acid Hydrolysis of Compounds **1**–**3** and GC Analysis

Compounds **1**–**3** (each 3 mg) were refluxed with 2 M HCl (1, 4 dioxane/H_2_O 1:1, 2 mL) on water bath for 2 h. After cooling, the reaction mixture was neutralized with 1 M NaOH. The reaction mixture was extracted with CHCl_3_ (3 × 5 mL). The aqueous layer was evaporated to dryness. The dried residue was dissolved in 1 mL anhydrous pyridine and treated with L-cysteine methyl ester hydrochloride (1.5 mg) stirred at 60 °C for 1 h. Trimethylsilylimidazole (1.0 mL) was added to the reaction mixtures, and they were kept at 60 °C for 30 min. The supernatants (4 μL) were analyzed by GC, respectively, under the following conditions: H_2_ flame ionization detector. Column: 30QC2/AC-5 quartz capillary column (30 m × 0.32 mm). Column temperature: 180–280 °C with the rate of 3 °C/min, and the carrier gas was N_2_ (1 mL/min) injector temperature: 250 °C; and split ratio: 1/50. Peaks of the hydrolysate were detected by comparison with retention times of authentic samples of D-glucose and D-xylose after treatment with trimethylchlorosilane (TMCS) in pyridine. The absolute configurations of the compounds **1**–**3** were determined by comparison of the retention times of the corresponding derivatives with those of standard D-glucose and D-xylose giving a single peak at 19.01 and 13.47 min, respectively.

### Cytotoxic Activity Assay

The following human cancer cell lines were used: T98G, U87, A549, GITC-3#, and GITC-18#. All cells were cultured in RPMI-1640 or DMEM medium (Hyclone, Logan, UT), supplemented with 10% fetal bovine serum (Hyclone) at 37 °C in a humidified atmosphere with 5% CO_2_. Cell viability was assessed by conducting colorimetric measurements of the amount of insoluble formazan formed in living cells based on the reduction of 3-(4,5-dimethylthiazol-2-yl)-2,5-diphenyltetrazolium bromide (MTT) (Sigma, St. Louis, MO) [31]. Briefly, 100 μL of adherent cells was seeded into each well of a 96-well cell culture plate and allowed to adhere for 12 h before drug addition, while suspended cells were seeded just before drug addition, both with an initial density of 1 × 10^5^ cells/mL in 100 μL of medium. Each cell line was exposed to the test compound at various concentrations in triplicate for 48 h, with cisplatin and paclitaxel (Sigma) as positive controls. After the incubation, MTT (100 μg) was added to each well, and the incubation continued for 4 h at 37 °C. The cells were lysed with 100 μL of 20% SDS-50% DMF after removal of 100 μL of medium. The optical density of the lysate was measured at 595 nm in a 96-well Microtiter plate reader (Bio-Rad 680).

## Electronic supplementary material

Below is the link to the electronic supplementary material.

Supplementary material 1 (PDF 3019 kb)
