# Supplementary material for: Indole Glycosides from Aqueous Fraction of Strychnos nitida
Source: Nat Prod Bioprospect. 2016 Nov 9;6(6):285–90. doi: 10.1007/s13659-016-0112-8 (PMC5136373; doi:10.1007/s13659-016-0112-8)

Supplementary data

Three New Indole Alkaloids from the Stem and Branch of *Strychnos nitida*

Bei Wang [a,b], Zhi Dai [b,c], Xin Wei [a,b], Pei-Feng Zhu [a,b], Hao-Fei Yu [a,b], Ya-Ping Liu [a,*], Xiao-Dong Luo [a,*]

a. State Key Laboratory of Phytochemistry and Plant Resources in West China, Kunming Institute of Botany Chinese Academy of Sciences, Kunming 650201, China.

b. University of Chinese Academy of Sciences, Beijing 100039, China.

c. Key Laboratory of Animal Models and Human Disease Mechanisms, Kunming Institute of Zoology, Chinese Academy of sciences, Kunming 650223, Yunnan, China.

Corresponding Authors

*xiao-dong luo

E-mail:xdluo@mail.kib.ac.cn

Tel: 86-871-65223177

Contents

Figures	Content
S1	Bioassay and General experimental procedures
S2	^{1}H NMR spectrum of compound **1** (CD_3OD).
S3	^{13}C and DEPT spectrum of compound **1** (CD_3OD).
S4	HMBC spectrum of compound **1** (CD_3OD).
S5	HSQC spectrum of compound **1** (CD_3OD)
S6	^{1}H-^{1}H COSY spectrum of compound **1** (CD_3OD).
S7	ROESY spectrum of compound **1** (CD_3OD).
S8	HRESIMS spectrum of compound **1**.
S9	IR spectrum of compound **1**.
S10	UV spectrum of compound **1**.
S11	ORD spectrum of compound **1**.
S12	^{1}H NMR spectrum of compound **2** (CD_3OD).
S13	^{13}C and DEPT spectrum of compound **2** (CD_3OD).
S14	HMBC spectrum of compound **2** (CD_3OD).
S15	HSQC spectrum of compound **2** (CD_3OD)
S16	^{1}H-^{1}H COSY spectrum of compound **2** (CD_3OD).
S17	ROESY spectrum of compound **2** (CD_3OD).
S18	HREIMS spectrum of compound **2**.
S19	IR spectrum of compound **2**.
S20	UV spectrum of compound **2**.
S21	ORD spectrum of compound **2**.
S22	^{1}H NMR spectrum of compound **3** (CD_3OD).
S23	^{13}C and DEPT spectrum of compound **3** (CD_3OD).
S24	HMBC spectrum of compound **3** (CD_3OD).
S25	HSQC spectrum of compound **3** (CD_3OD)
S26	^{1}H-^{1}H COSY spectrum of compound **3** (CD_3OD).
S27	ROESY spectrum of compound **3** (CD_3OD).

S28	HRESIMS spectrum of compound **3**.
S29	IR spectrum of compound **3**.
S30	UV spectrum of compound **3**.
S31	ORD spectrum of compound **3**.
S32	HSQC-TOCSY spectrum of compound **3**.

S1 Bioassay and General experimental procedures

S1.1 Cytotoxic Activity Assay

The following human cancer cell lines were used: SW-480, SMMC-7721, HL-60, MCF-7, and A-549. All cells were cultured in RPMI-1640 or DMEM medium (Hyclone, Logan, UT), supplemented with 10 % fetal bovine serum (Hyclone) at 37°C in a humidified atmosphere with 5 % CO_2. Cell viability was assessed by conducting colorimetric measurements of the amount of insoluble formazan formed in living cells based on the reduction of 3-(4, 5-dimethylthiazol-2-yl)-2, 5-diphenyltetrazolium bromide (MTT) (Sigma, St. Louis, MO) . Briefly, 100 μLof adherent cells was seeded into each well of a 96-well cell culture plate and allowed to adhere for 12 h before drug addition, while suspended cells were seeded just before drug addition, both with an initial density of 1×10^5 cells/mL in 100 μL of medium. Each cell line was exposed to the test compound at various concentrations in triplicate for 48 h, with cisplatin and paclitaxel (Sigma) as positivecontrols. After the incubation, MTT (100 μg) was added to each well, and the incubation continued for 4 h at 37 °C. The cells were lysed with 100 μL of 20% SDS-50% DMF after removal of 100 μL of medium. The optical density of the lysate was measured at 595 nm in a 96-well Microtiter plate reader (Bio-Rad 680).

S1.2 General experimental procedures

Optical rotations were obtained with a Jasco P-1020 Automatic Digital Polariscope. UV spectrum was measured with a Shi madzu UV2401PC spectrometer. IR spectra were obtained on a Bruker FT-IR Tensor-27 infrared spectrophotometer with KBr pellets. ^{1}H, ^{13}C, and 2D NMR spectra were recorded on a Bruker DRX-400 NMR, Bruker DRX-500 NMR and Bruker DRX-600 spectrometer with TMS as internal standard. ESI-MS and HR-EI-MS analysis were carried out on Waters Xevo TQS and Waters AutoSpec Premier P776 mass spectrometers, respectively. Semi-preparative HPLC was performed on an Waters 600 HPLC with a COSMOSIL 5C_{18} MS-II (10ID × 250 mm) column. Column chromatography (CC) was performed on silica gel (100–200 and 200–300 mesh, Qingdao Marine Chemical Co. Ltd., P.R. China), Sephadex LH-20 (GE Healthcare Bio-Xciences AB), and MCI gel (75–150 lm, Mitsubishi Chemical

Corporation, Tokyo, Japan) were used for column chromatography. RP-18 gel (20–45 lm, Fuji Silysia Chemical Ltd., Japan), and Sephadex LH-20 (GE Healthcare Bio-sciences AB, Sala, Sweden). Fractions were monitored by TLC (GF 254, Qingdao Marine Chemical Co., Ltd., Qingdao), and spots were visualized by Dragendorff's reagent. GC analysis was performed on a Shimadzu GC-2010 gas chromatograph equipped with an H_2 flame ionization detector. D-xylose and D-glucose were purchased from J K Scientific Ltd. (Guangzhou, China).

Figure S2. ^{1}H NMR spectrum of compound **1** (CD_3OD).

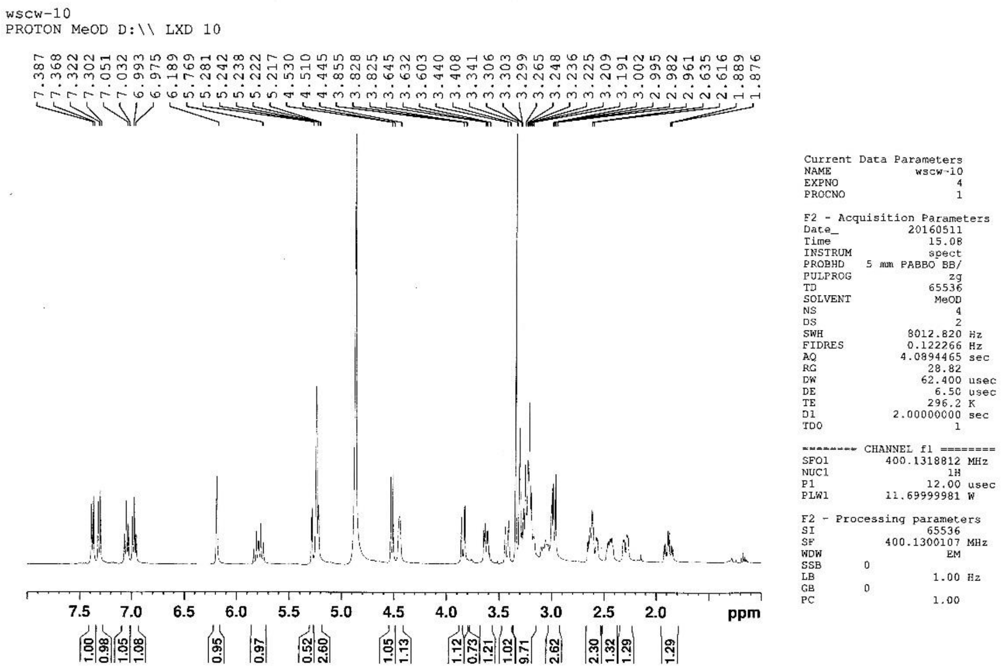

Figure S3. ^{13}C and DEPT spectrum of compound **1** (CD_3OD).

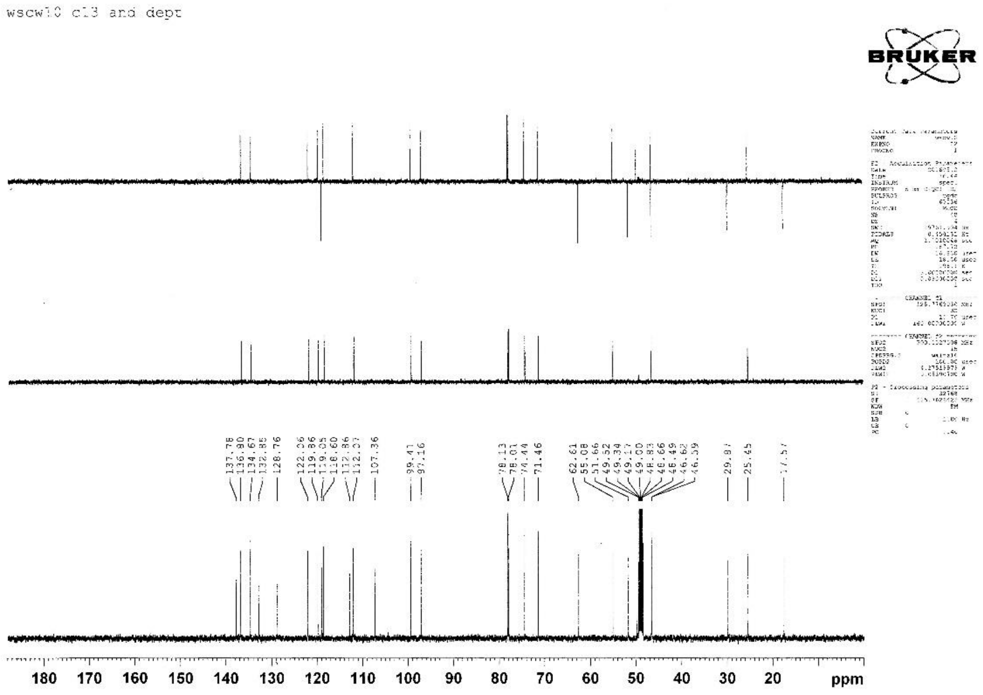

Figure S4. HMBC spectrum of compound **1** (CD_3OD) .

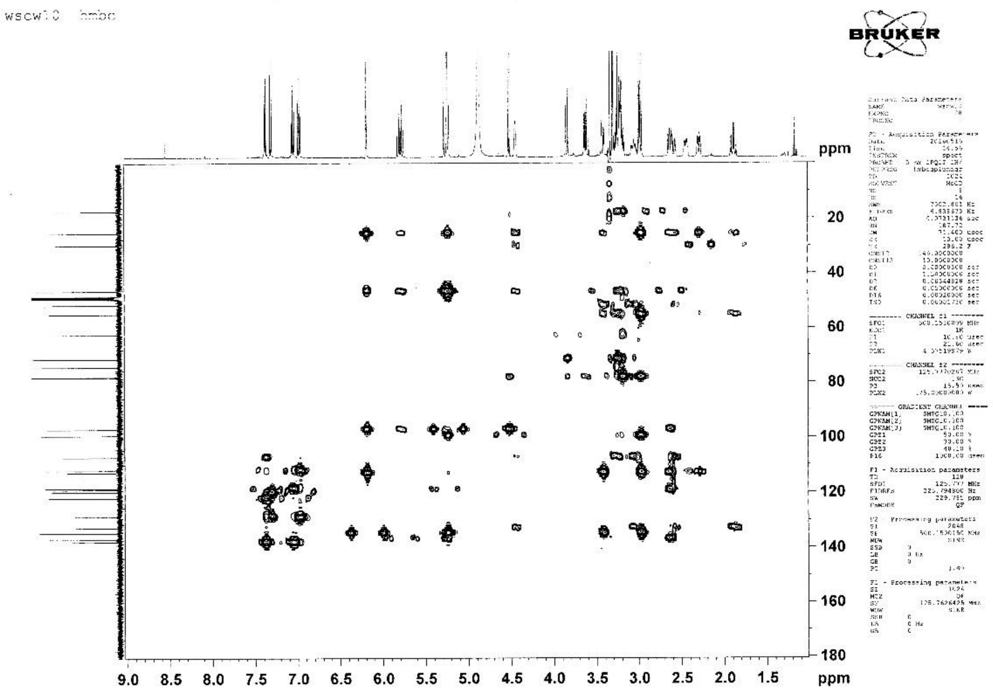

Figure S5. HSQC spectrum of compound **1** (CD_3OD).

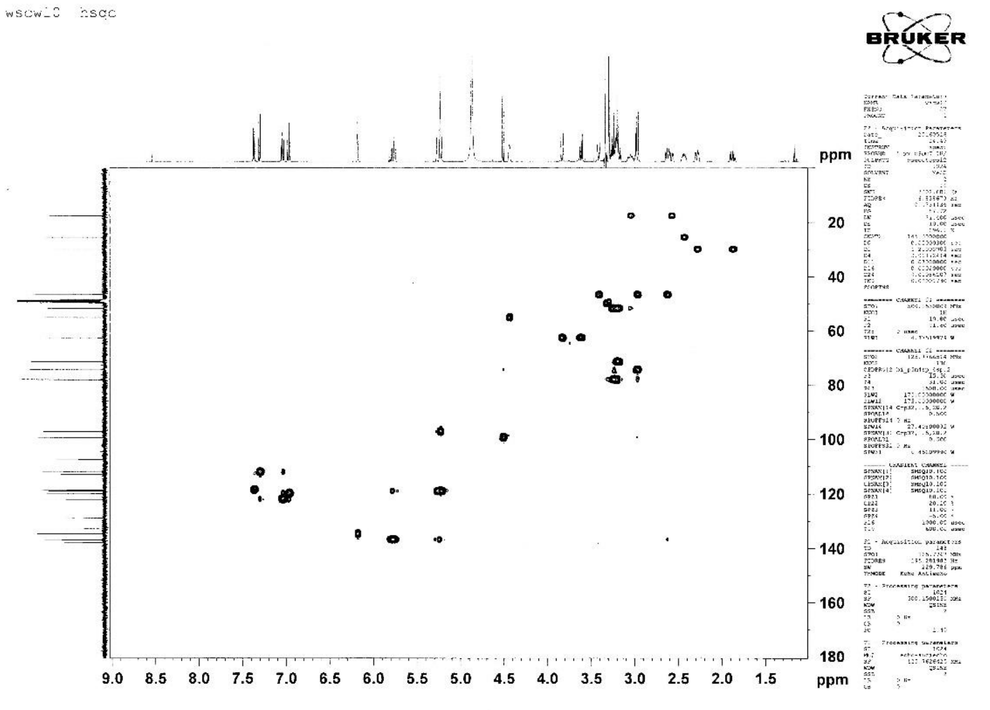

Figure S6. ^{1}H-^{1}H COSY spectrum of compound **1** (CD_3OD).

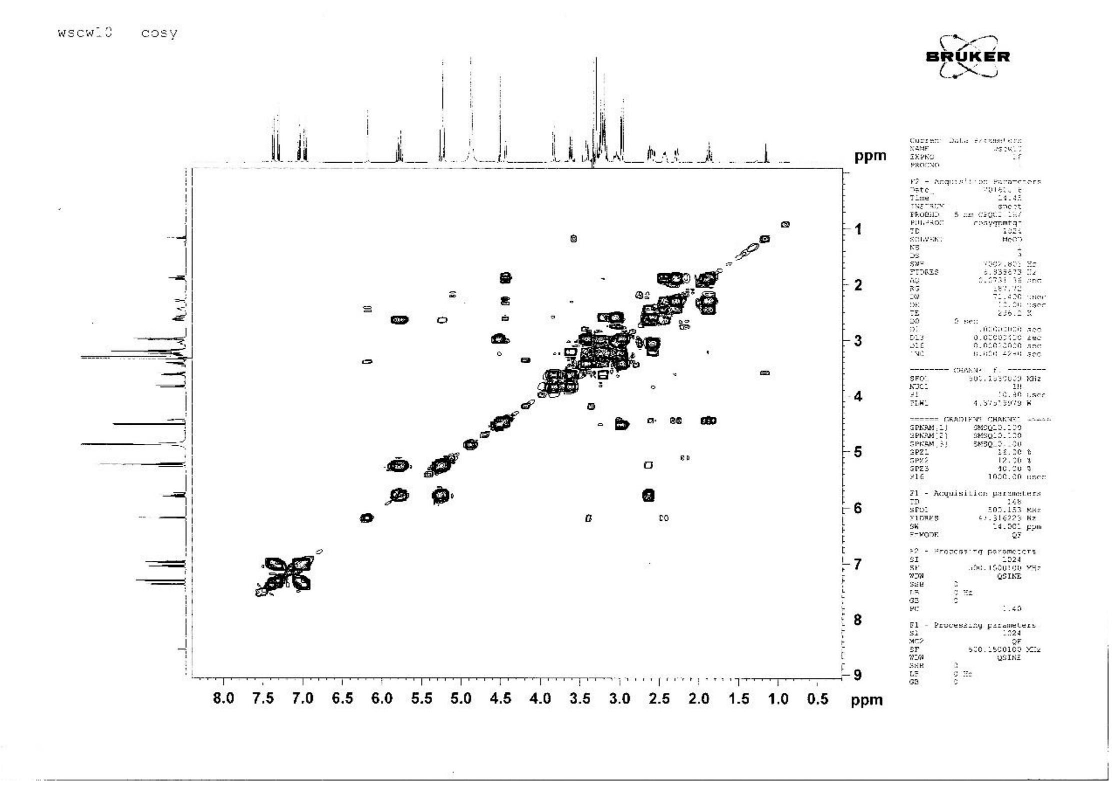

Figure S7. ROESY spectrum of compound **1** (CD_3OD).

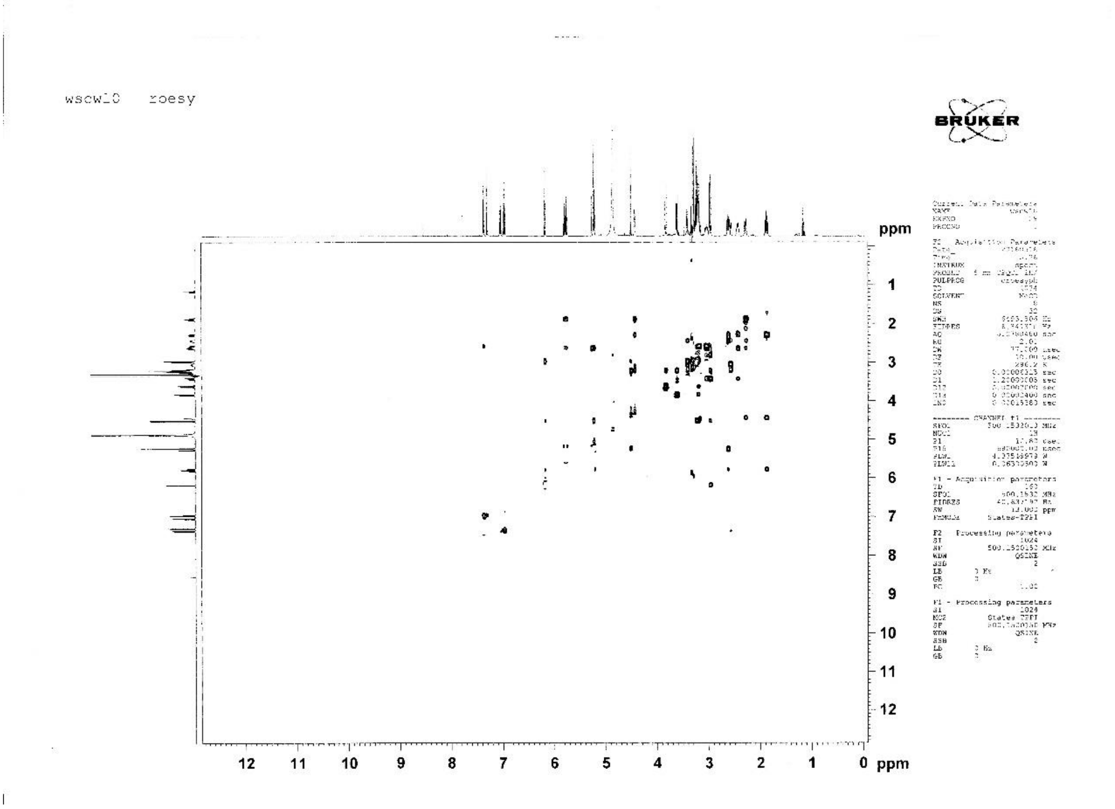

Figure S8. HRESIMS spectrum of compound **1**.

Qualitative Analysis Report

**Data Filename**	wscw-10+.d	**Sample Name**	wscw-10
**Sample Type**	Sample	**Position**	P1-E1
**Instrument Name**	Instrument 1	**User Name**	
**Acq Method**	SIBU.m	**Acquired Time**	5/19/2016 1:39:19 PM
**IRM Calibration Status**	[redacted]	**DA Method**	ESI+.m
**Comment**			

Sample Group **Info.**
Acquisition SW Version 6200 series TOF/6500 series Q-TOF B.05.01 (B5125.2)

User Spectra

Fragmentor Voltage	Collision Energy	Ionization Mode
135	0	ESI

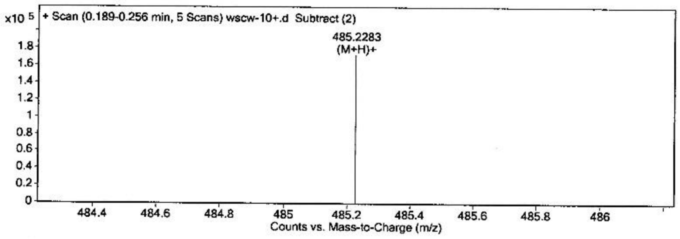

Peak List

m/z	z	Abund	Formula	Ion
311.2109	1	2823.32		
453.167	1	2274.12		
485.2283	1	173466.61	C26 H32 N2 O7	(M+H)+
486.2314	1	47801.32	C26 H32 N2 O7	(M+H)+
487.2342	1	8921.01	C26 H32 N2 O7	(M+H)+
501.223	1	12743.76		
502.226	1	3637.11		
523.1845	1	2774.23		

Formula Calculator Element Limits

Element	Min	Max
C	3	120
H	0	240
O	0	100
N	0	100

Formula Calculator Results

Formula	CalculatedMass	CalculatedMz	Mz	Diff. (mDa)	Diff. (ppm)	DBE
C26 H32 N2 O7	484.2210	485.2282	485.2283	-0.1	-0.1	12.0000

--- End Of Report ---

Agilent Technologies Page 1 of 1 Printed at: 1:44 PM on: 5/19/2016

Figure S9. IR spectrum of compound **1**.

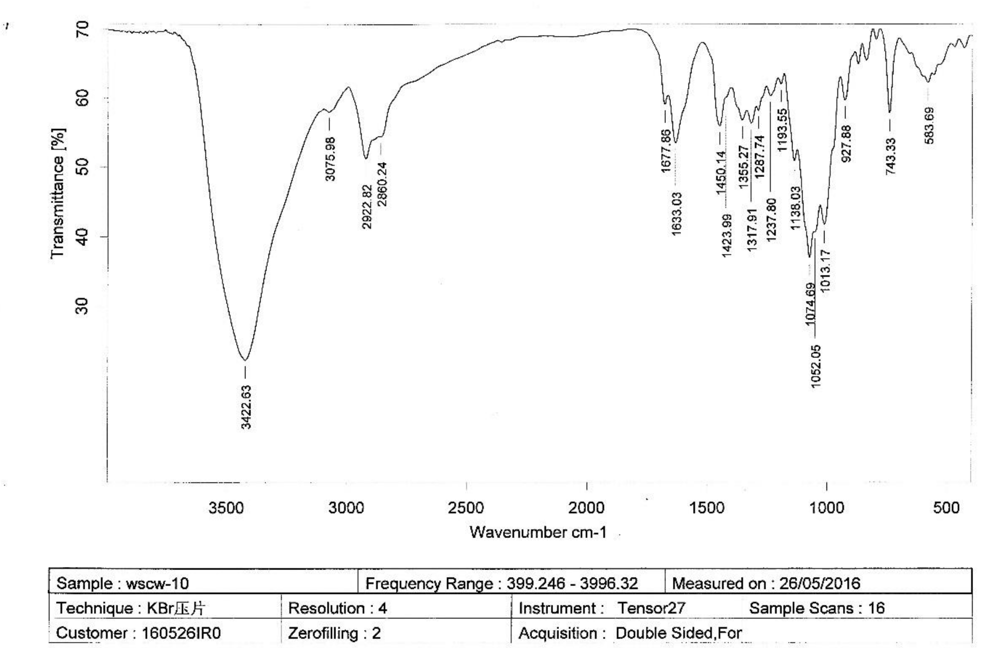

Figure S10. UV spectrum of compound **1**.

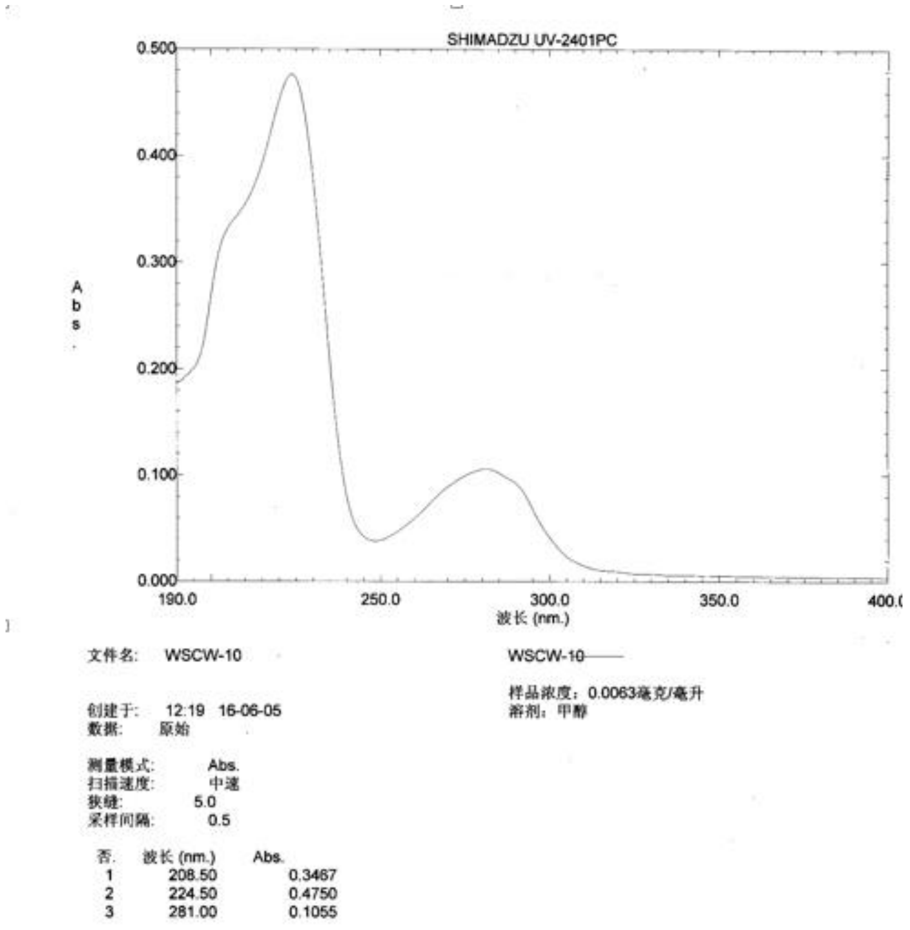

Figure S11. ORD spectrum of compound **1**.

Optical rotation measurement

Model : P-1020 (A060460638)

No.	Sample	Mode	Data	Monitor<br>Blank	Temp.<br>Cell<br>Temp Point	Date<br>Comment<br>Sample Name	Light<br>Filter<br>Operator	Cycle Time<br>Integ Time
No.1	3 (1/3)	Sp.Rot	-78.6670	-0.0118<br>0.0000	24.6<br>10.00<br>Cell	Fri Jun 03 17:48:58 2016<br>0.00150g/mL MeOH<br>WSCW-10	Na<br>589nm	2 sec<br>10 sec
No.2	3 (2/3)	Sp.Rot	-84.0000	-0.0126<br>0.0000	24.6<br>10.00<br>Cell	Fri Jun 03 17:49:11 2016<br>0.00150g/mL MeOH<br>WSCW-10	Na<br>589nm	2 sec<br>10 sec
No.3	3 (3/3)	Sp.Rot	-77.3330	-0.0116<br>0.0000	24.6<br>10.00<br>Cell	Fri Jun 03 17:49:25 2016<br>0.00150g/mL MeOH<br>WSCW-10	Na<br>589nm	2 sec<br>10 sec
No.4	4 (1/3)	Sp.Rot	-80.0000	-0.0120<br>0.0000	24.6<br>10.00<br>Cell	Fri Jun 03 17:49:52 2016<br>0.00150g/mL MeOH<br>WSCW-10	Na<br>589nm	2 sec<br>10 sec
No.5	4 (2/3)	Sp.Rot	-76.6670	-0.0115<br>0.0000	24.6<br>10.00<br>Cell	Fri Jun 03 17:50:06 2016<br>0.00150g/mL MeOH<br>WSCW-10	Na<br>589nm	2 sec<br>10 sec
No.6	4 (3/3)	Sp.Rot	-78.6670	-0.0118<br>0.0000	24.6<br>10.00<br>Cell	Fri Jun 03 17:50:19 2016<br>0.00150g/mL MeOH<br>WSCW-10	Na<br>589nm	2 sec<br>10 sec

-78.3333°

Figure S12. ^{1}H NMR spectrum of compound **2** (CD_3OD).

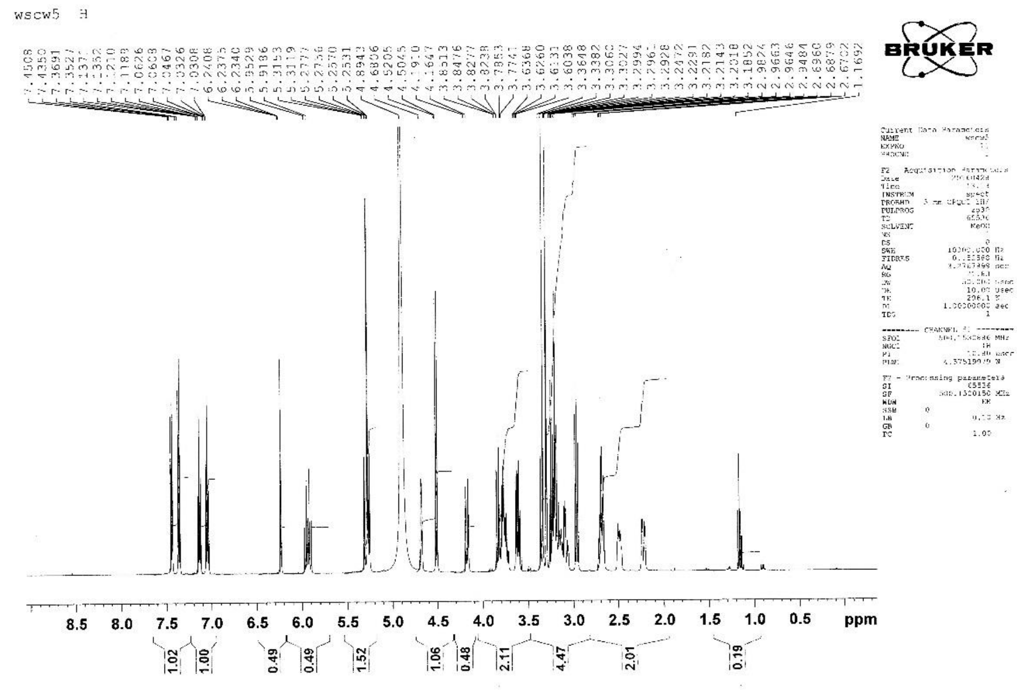

Figure S13.^{13}C and DEPT spectrum of compound **2** (CD_3OD).

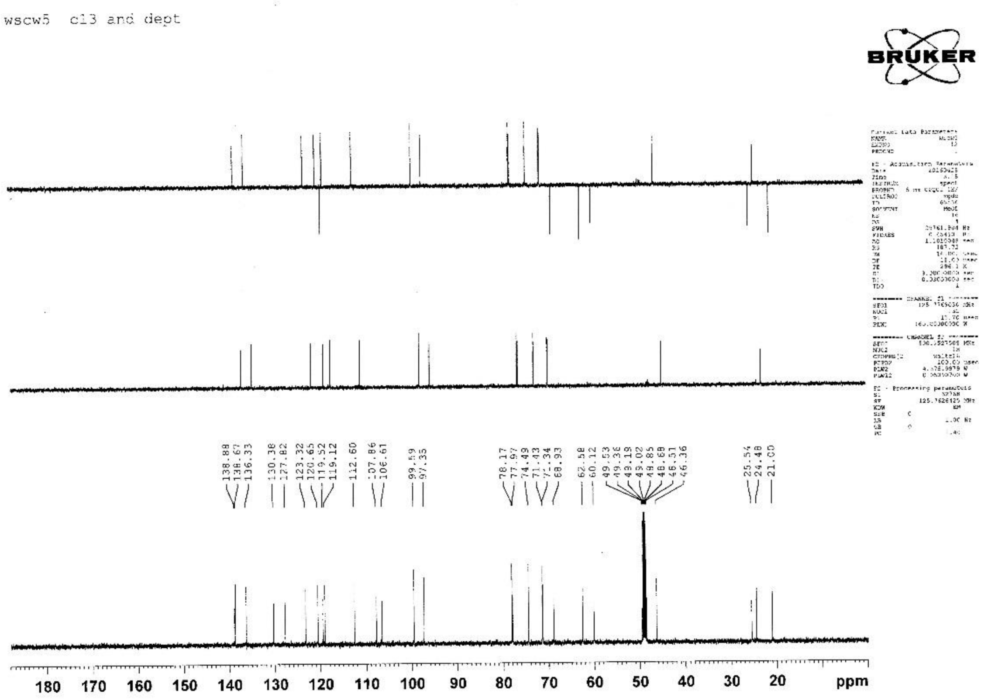

Figure S14. HMBC spectrum of compound **2** (CD_3OD).

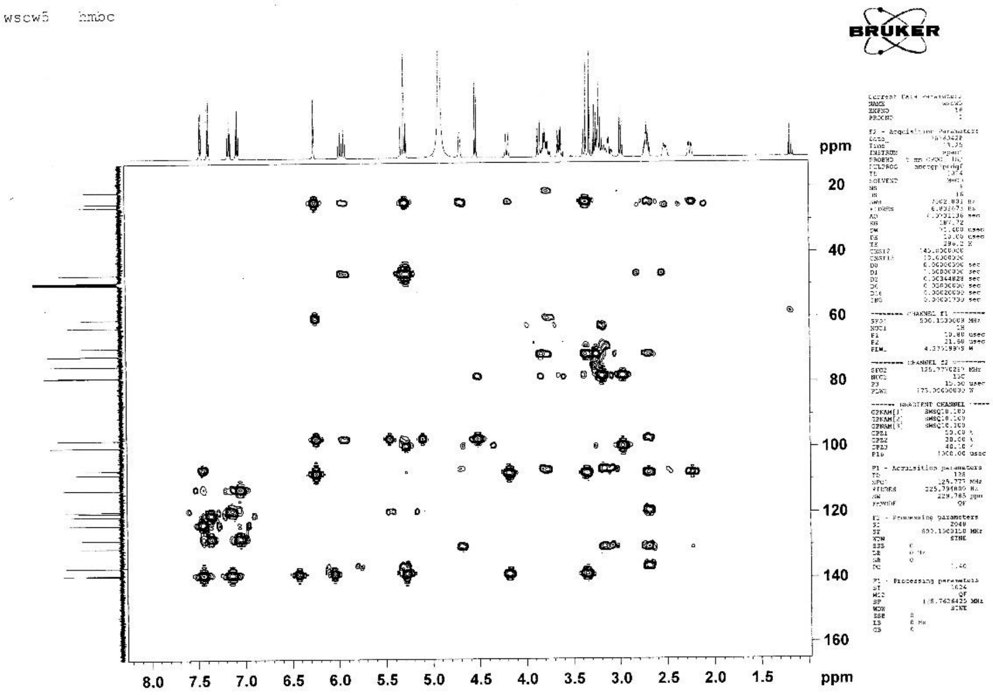

Figure S15. HSQC spectrum of compound **2**(CD_3OD)

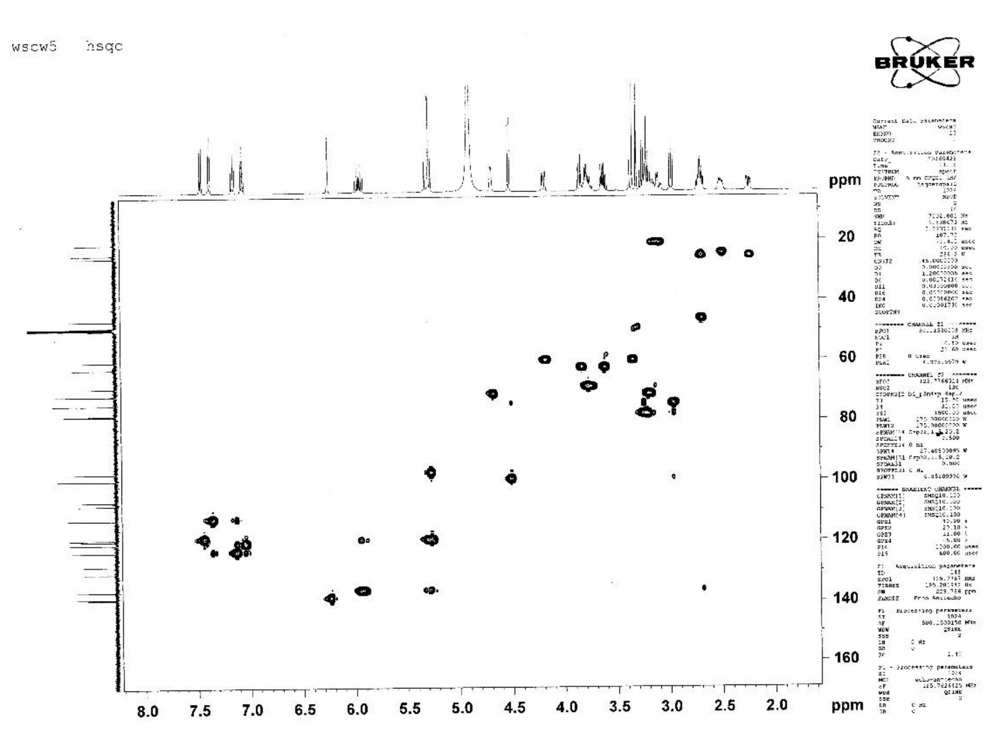

Figure S16. ^{1}H-^{1}H COSY spectrum of compound **2** (CD_3OD).

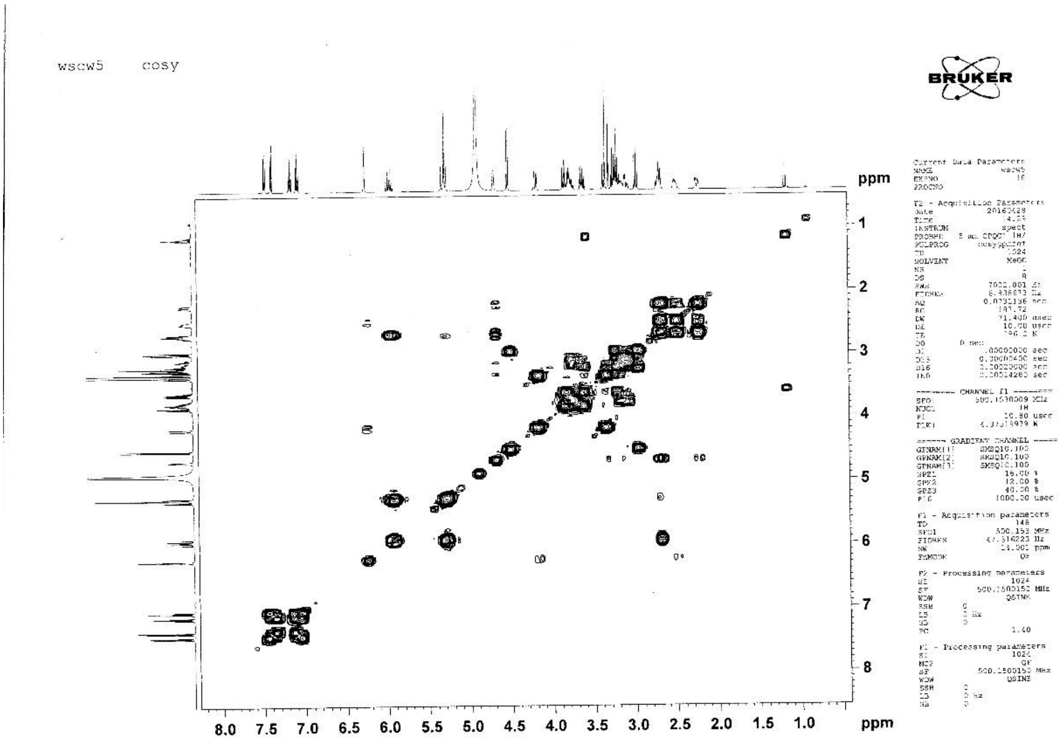

Figure S17. ROESY spectrum of compound **2** (CD_3OD).

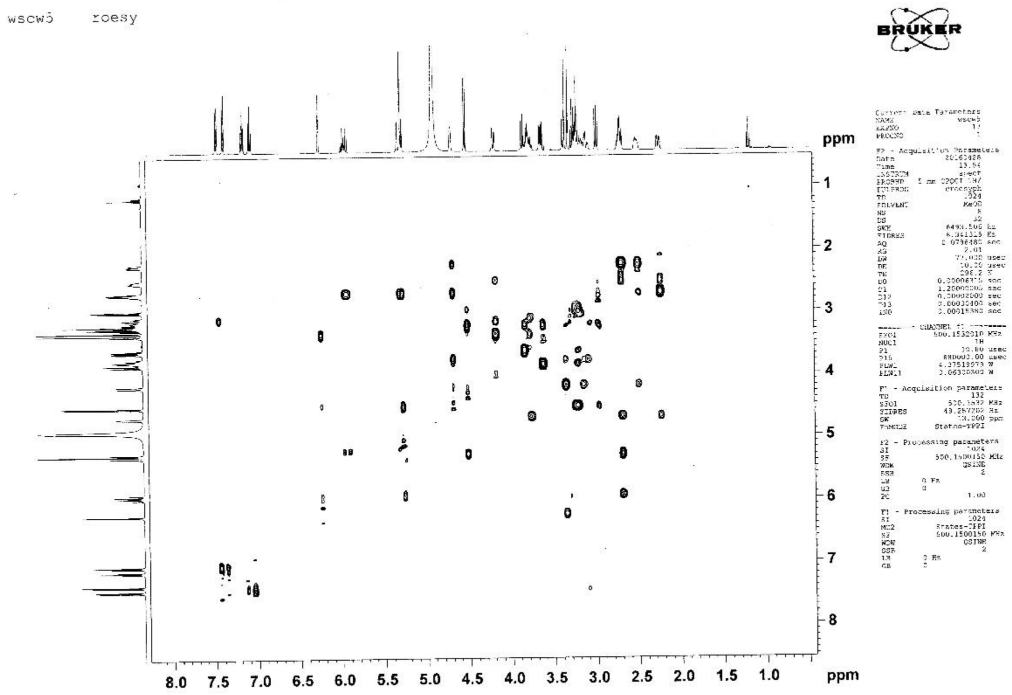

Figure S18. HREIMS spectrum of compound **2**.

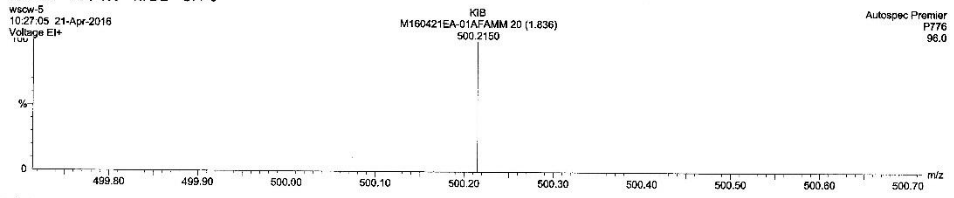

Minimum:				-10.0		
Maximum:		200.0	10.0	120.0		
Mass	Calc. Mass	mDa	PPM	DBE	i-FIT	Formula
500.2150	500.2159	-0.9	-1.8	12.0	5546065.5	C26 H32 N2 O8

Figure S19. IR spectrum of compound **2**.

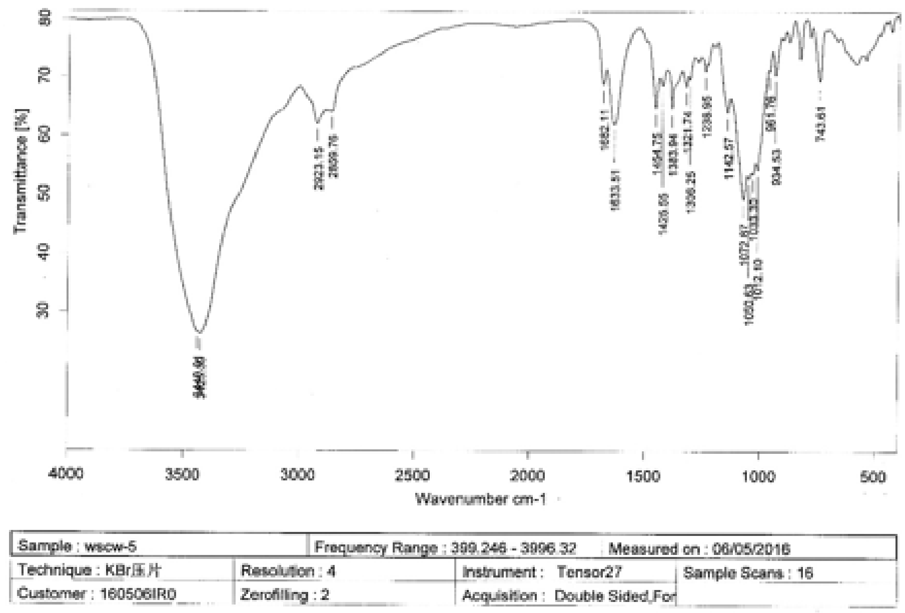

Figure S20. UV spectrum of compound **2**.

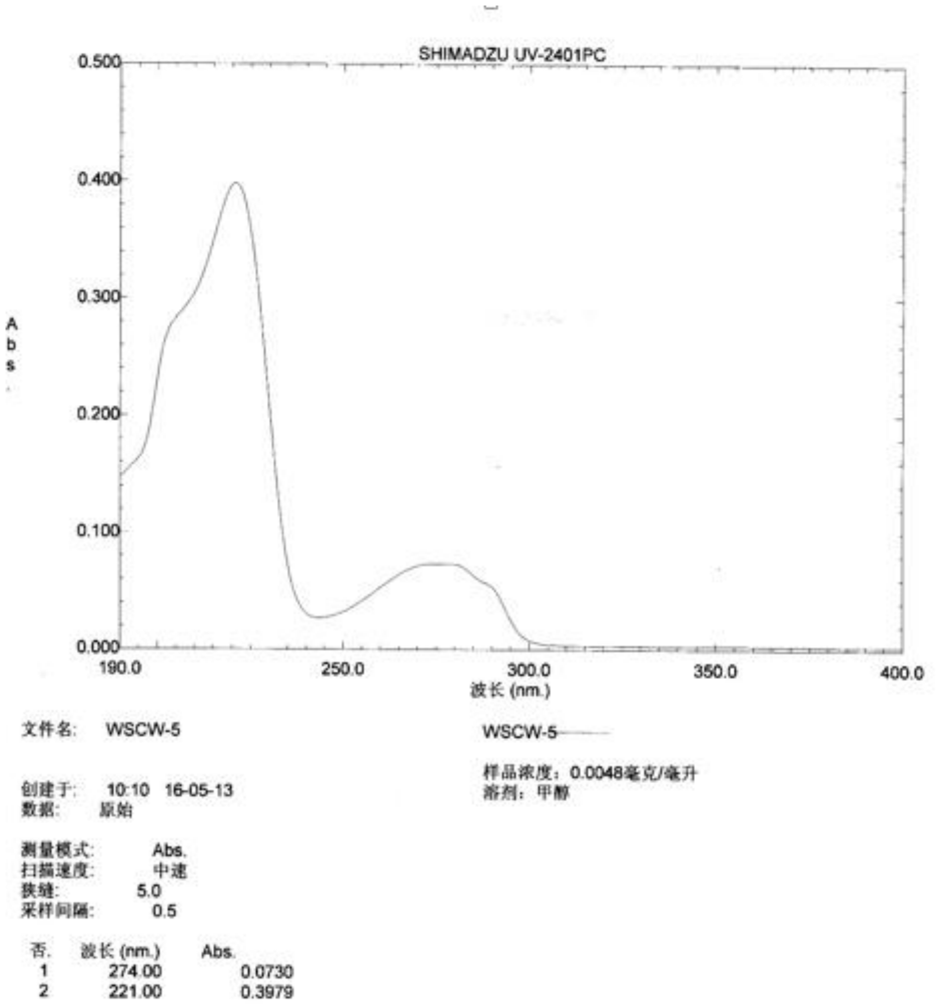

Figure S21. ORD spectrum of compound **2**.

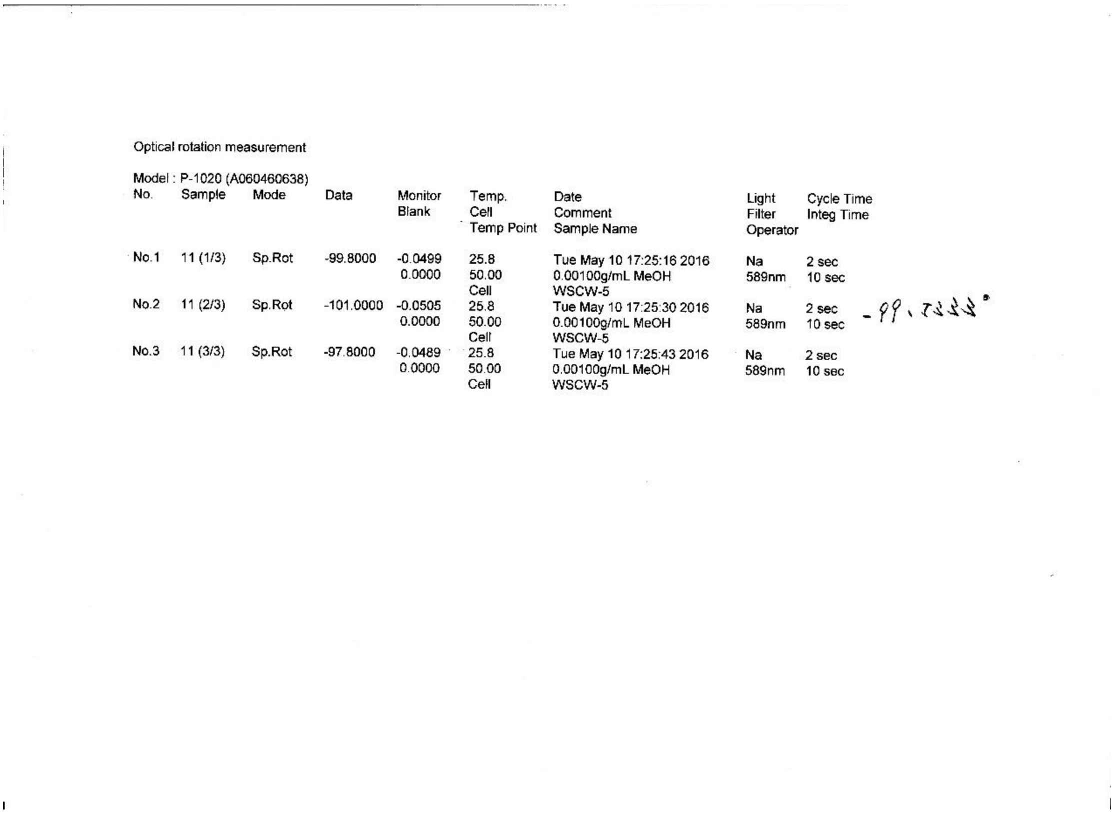

Optical rotation measurement

Model : P-1020 (A060460638)

No.	Sample	Mode	Data	Monitor<br>Blank	Temp.<br>Cell<br>Temp Point	Date<br>Comment<br>Sample Name	Light<br>Filter<br>Operator	Cycle Time<br>Integ Time
No.1	11 (1/3)	Sp.Rot	-99.8000	-0.0499<br>0.0000	25.8<br>50.00<br>Cell	Tue May 10 17:25:16 2016<br>0.00100g/mL MeOH<br>WSCW-5	Na<br>589nm	2 sec<br>10 sec
No.2	11 (2/3)	Sp.Rot	-101.0000	-0.0505<br>0.0000	25.8<br>50.00<br>Cell	Tue May 10 17:25:30 2016<br>0.00100g/mL MeOH<br>WSCW-5	Na<br>589nm	2 sec<br>10 sec
No.3	11 (3/3)	Sp.Rot	-97.8000	-0.0489<br>0.0000	25.8<br>50.00<br>Cell	Tue May 10 17:25:43 2016<br>0.00100g/mL MeOH<br>WSCW-5	Na<br>589nm	2 sec<br>10 sec

-99.5333°

Figure S22. ^{1}H NMR spectrum of compound **3** (CD_3OD).

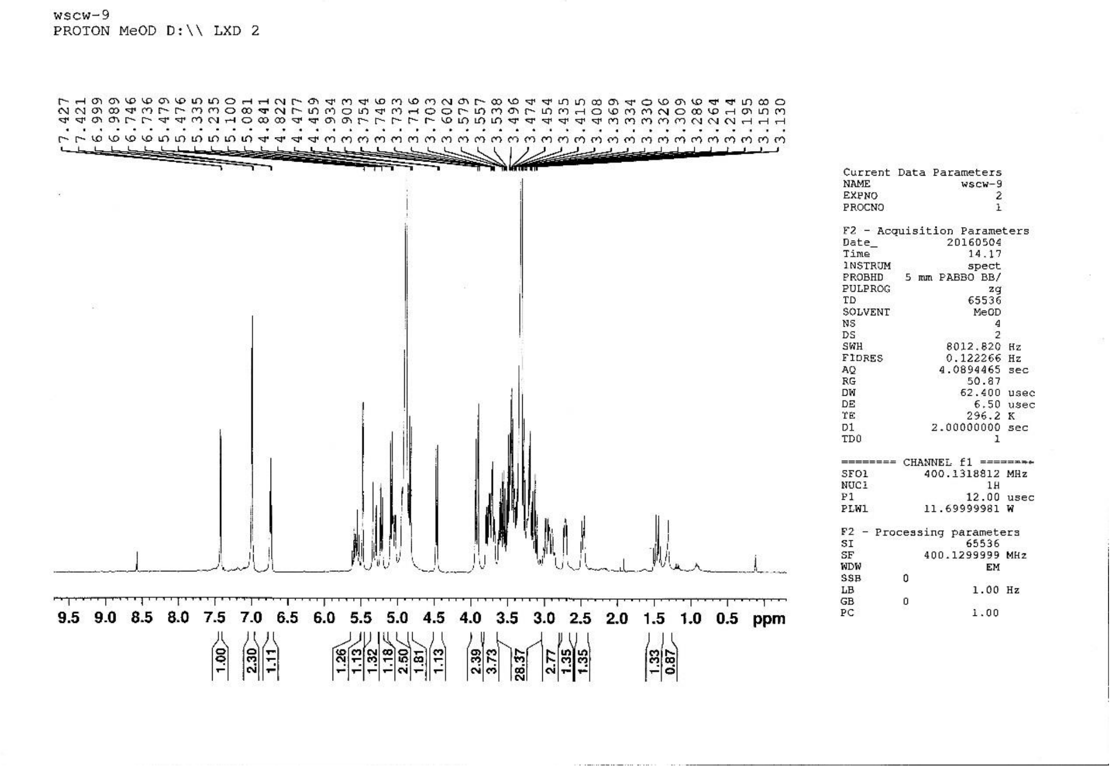

Figure S23.^{13}C and DEPT spectrum of compound **3** (CD_3OD).

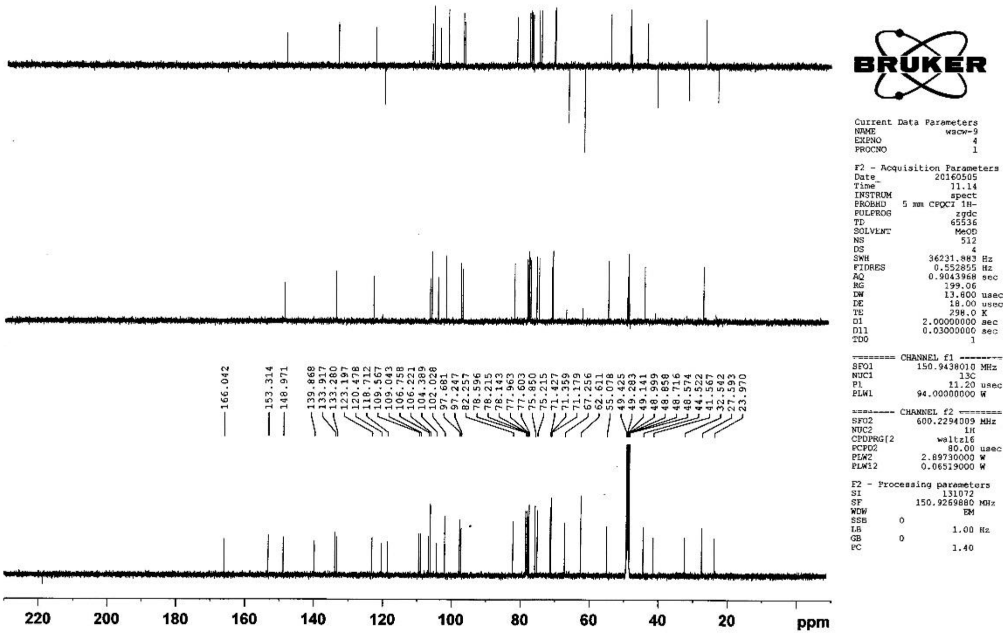

Figure S24. HMBC spectrum of compound **3** (CD_3OD).

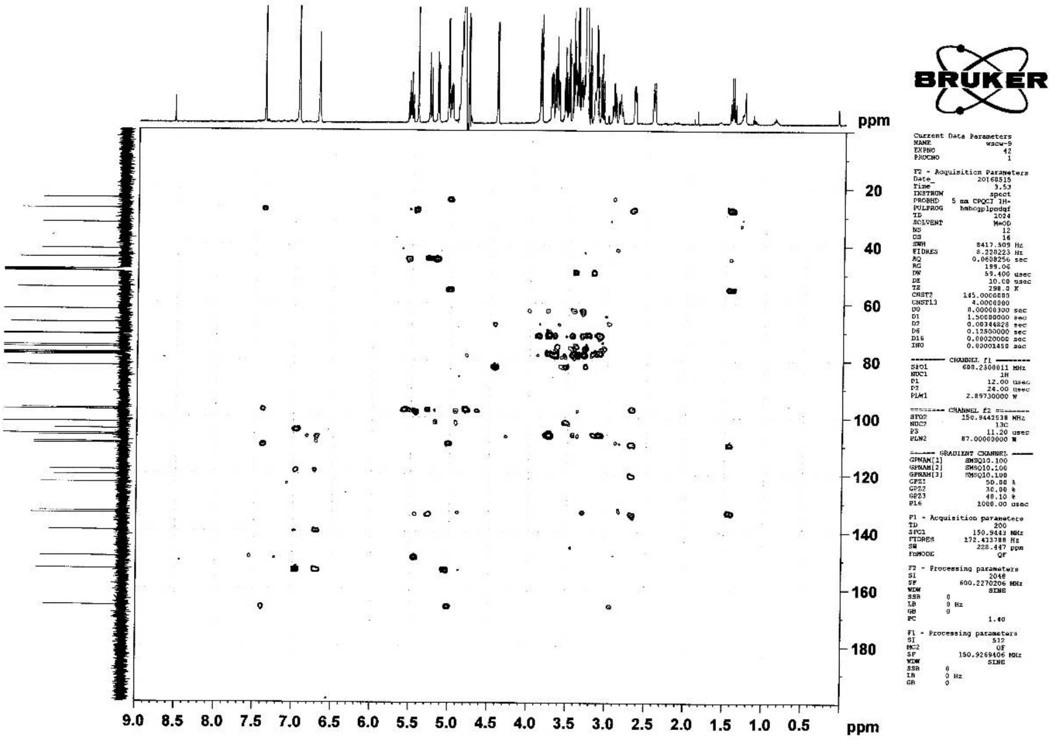

Figure S25. HSQC spectrum of compound **3**(CD_3OD)

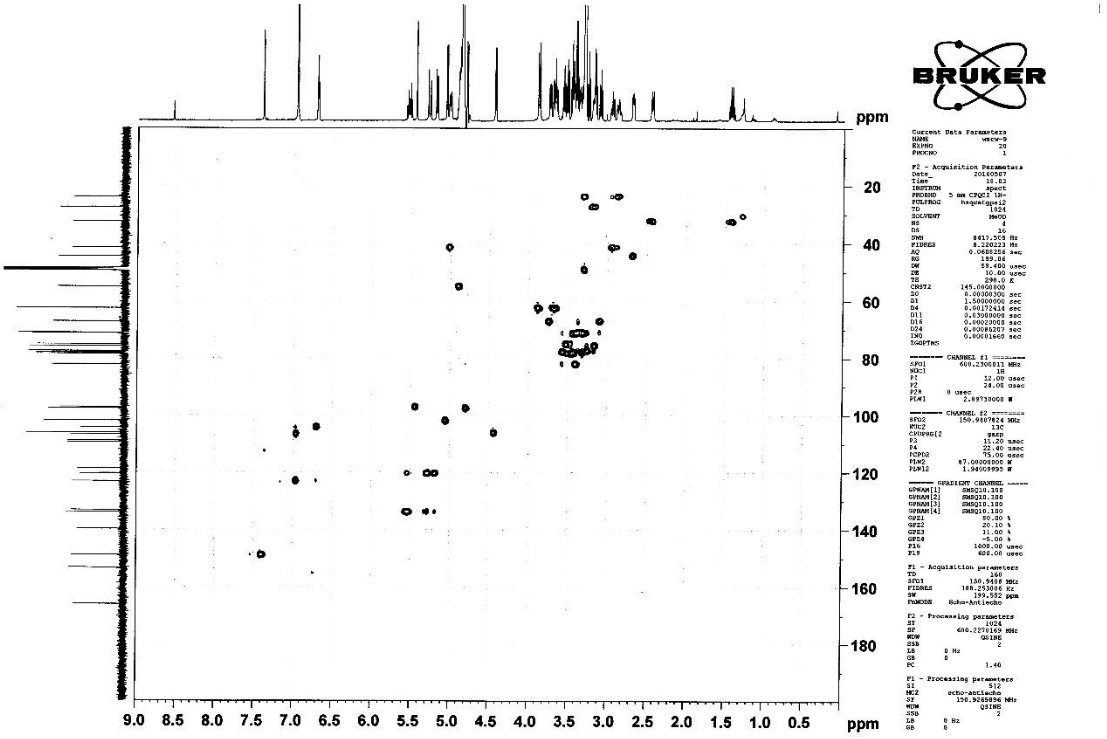

Figure S26. ^{1}H-^{1}H COSY spectrum of compound **3** (CD_3OD).

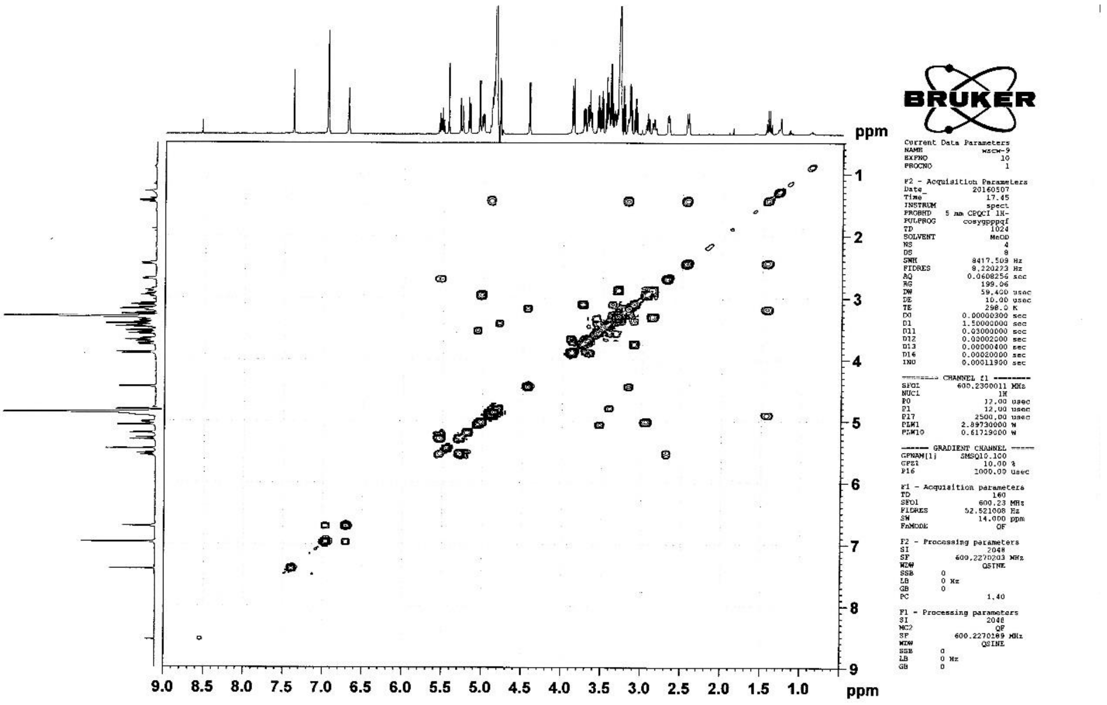

Figure S27. ROESY spectrum of compound **3** (CD_3OD).

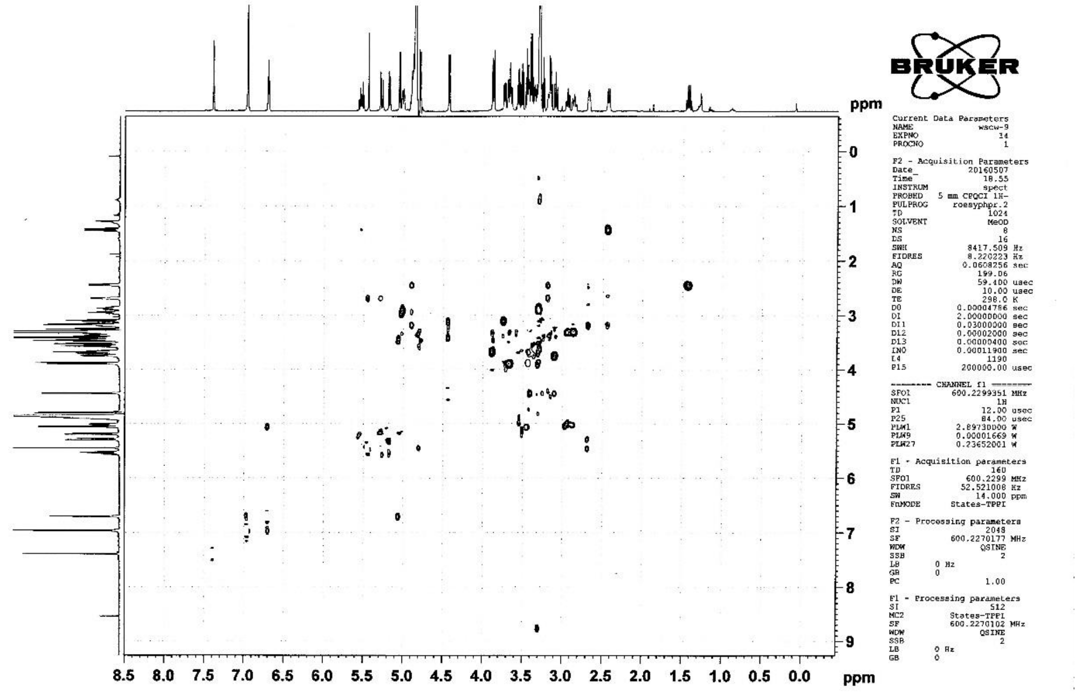

igure S28. HRESIMS spectrum of compound **3**.

Qualitative Analysis Report

**Data Filename**	wscw-9.d	**Sample Name**	wscw-9
**Sample Type**	Sample	**Position**	P1-C6
**Instrument Name**	Instrument 1	**User Name**	
**Acq Method**	SIBU.m	**Acquired Time**	5/13/2016 9:56:47 AM
**IRM Calibration Status**	[redacted]	**DA Method**	ESI+.m
**Comment**			

Sample Group **Info.**
Acquisition SW Version 6200 series TOF/6500 series Q-TOF B.05.01 (B5125.2)

User Spectra

Fragmentor Voltage	Collision Energy	Ionization Mode
135	0	ESI

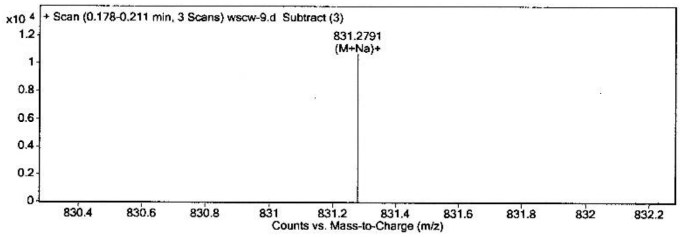

Peak List

m/z	z	Abund	Formula	Ion
311.2119	1	34561.29		
312.2172	1	7136.46		
424.1266	2	8459.9		
512.504		4149.75		
809.2971	1	7403.3		
831.2791	1	10727.56	C37 H48 N2 O18	(M+Na)+
832.2815	1	4176.21	C37 H48 N2 O18	(M+Na)+
955.5692	1	4782		

Formula Calculator Element Limits

Element	Min	Max
C	3	60
H	0	120
O	0	30
N	0	5

Formula Calculator Results

Formula	CalculatedMass	CalculatedMz	Mz	Diff. (mDa)	Diff. (ppm)	DBE
C37 H48 N2 O18	808.2902	831.2794	831.2791	0.6	0.8	15.0000

--- End Of Report ---

Agilent Technologies Page 1 of 1 Printed at: 1:17 PM on: 5/13/2016

Figure S29. IR spectrum of compound **3**.

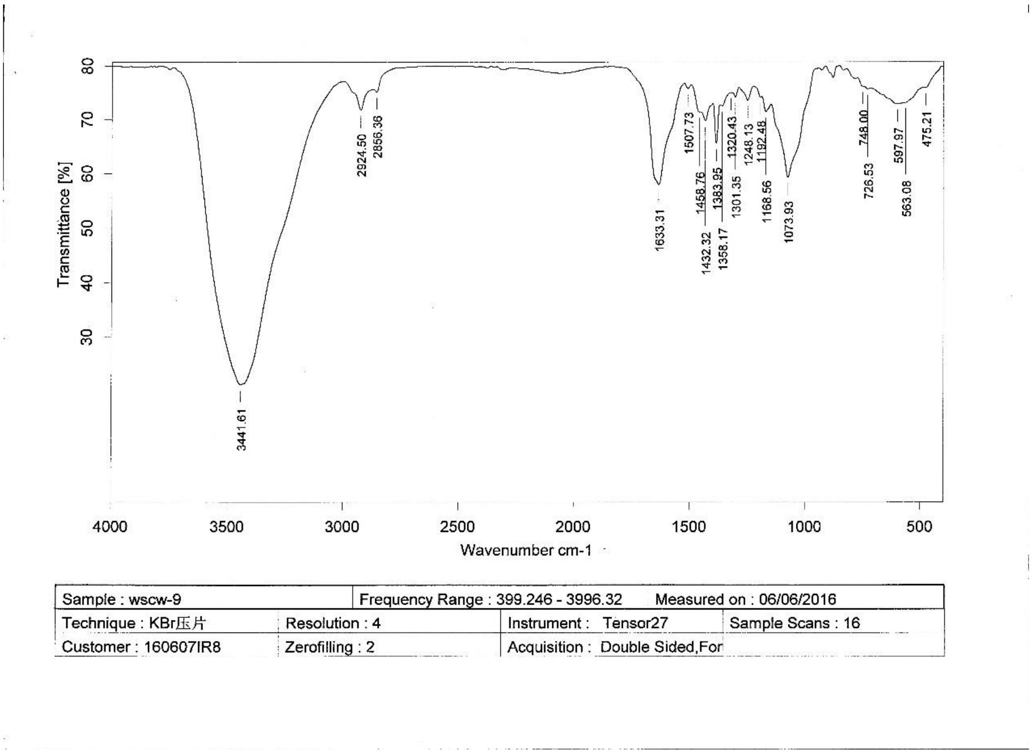

Figure S30. UV spectrum of compound **3**.

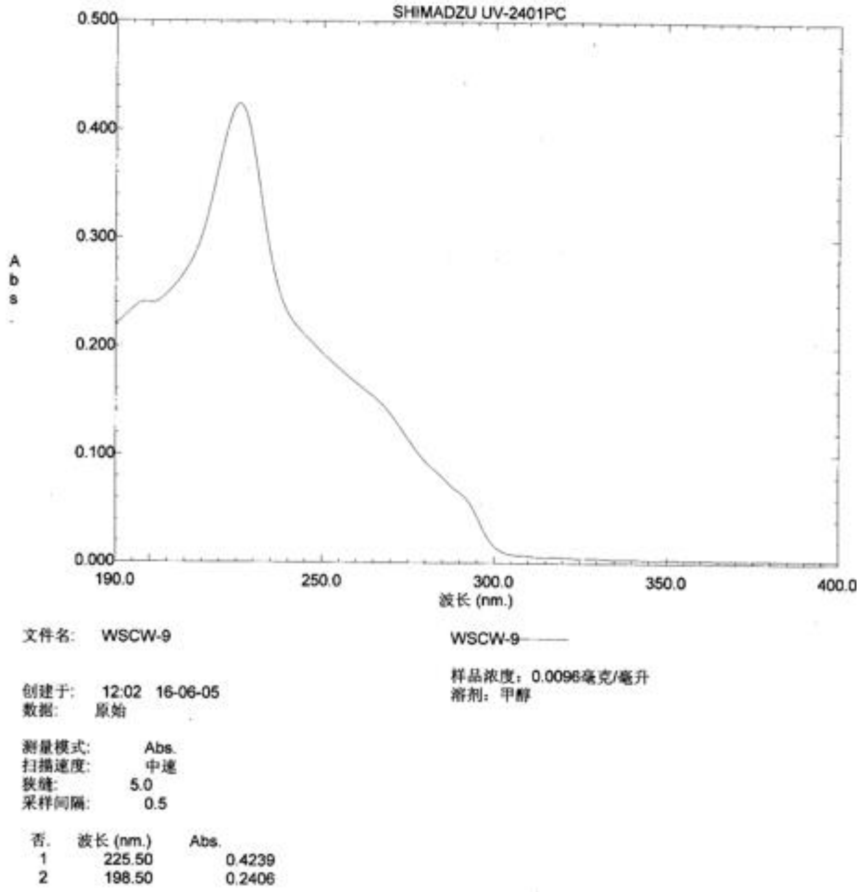

Figure S31. ORD spectrum of compound **3**.

Optical rotation measurement

Model : P-1020 (A060460638)

No.	Sample	Mode	Data	Monitor<br>Blank	Temp.<br>Cell<br>Temp Point	Date<br>Comment<br>Sample Name	Light<br>Filter<br>Operator	Cycle Time<br>Integ Time
No.1	5 (1/3)	Sp.Rot	-115.0000	-0.0115<br>0.0000	24.6<br>10.00<br>Cell	Fri Jun 03 17:59:31 2016<br>0.00100g/mL MeOH<br>WSCW-9	Na<br>589nm	2 sec<br>10 sec
No.2	5 (2/3)	Sp.Rot	-119.0000	-0.0119<br>0.0000	24.6<br>10.00<br>Cell	Fri Jun 03 17:59:45 2016<br>0.00100g/mL MeOH<br>WSCW-9	Na<br>589nm	2 sec<br>10 sec
No.3	5 (3/3)	Sp.Rot	-125.0000	-0.0125<br>0.0000	24.6<br>10.00<br>Cell	Fri Jun 03 17:59:58 2016<br>0.00100g/mL MeOH<br>WSCW-9	Na<br>589nm	2 sec<br>10 sec
No.4	6 (1/3)	Sp.Rot	-112.0000	-0.0112<br>0.0000	24.7<br>10.00<br>Cell	Fri Jun 03 18:01:11 2016<br>0.00100g/mL MeOH<br>WSCW-9	Na<br>589nm	2 sec<br>10 sec
No.5	6 (2/3)	Sp.Rot	-114.0000	-0.0114<br>0.0000	24.7<br>10.00<br>Cell	Fri Jun 03 18:01:25 2016<br>0.00100g/mL MeOH<br>WSCW-9	Na<br>589nm	2 sec<br>10 sec
No.6	6 (3/3)	Sp.Rot	-121.0000	-0.0121<br>0.0000	24.6<br>10.00<br>Cell	Fri Jun 03 18:01:38 2016<br>0.00100g/mL MeOH<br>WSCW-9	Na<br>589nm	2 sec<br>10 sec

-117.6667°

Figure S32. HSQC-TOCSY spectrum of compound **3**.

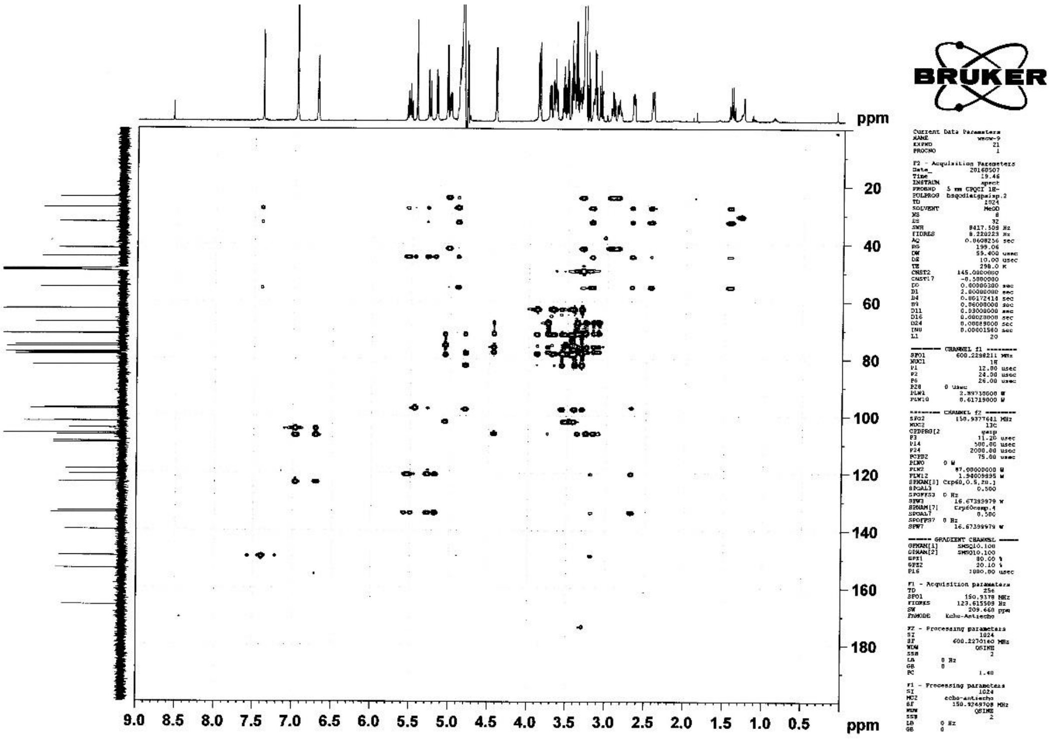

Supplement: Supplementary file 1 — Supplementary material 1 (PDF 3019 kb) [file 13659_2016_112_MOESM1_ESM.pdf]
